# Antimutagenic and Antiapoptotic Effects of Aqueous Root Extract of *Inula racemosa* Hook. f. on 4-NQO-Induced Genetic Damage in Mice

**DOI:** 10.1155/2013/768359

**Published:** 2013-09-03

**Authors:** P. Arumugam, M. Murugan

**Affiliations:** ^1^Centre for Advanced Studies in Botany, School of Life Science, University of Madras, Guindy, Chennai, Tamil Nadu 600 113, India; ^2^Department of Microbial Technology, School of Biological Science, Madurai Kamaraj University, Madurai, Tamil Nadu 625 021, India

## Abstract

The present study was performed as part of an attempt to authenticate the use of *Inula racemosa* root extract as traditional medicine in India by experimentally investigating their protective effects on 4-nitroquinoline-1-oxide (4-NQO) induced DNA damage and apoptosis in mice bone marrow cells. Aqueous root extract (ARE) of *Inula racemosa* (100, 200 and 400 mg/kg bw) with and without 4-NQO along with vehicle control (H_2_O) were administered orally for five consecutive days. 4-NQO (7.5 mg/kg bw) was injected intraperitoneally to the mice on the sixth day. After 24 h, the animals were sacrificed and extracted bone marrow cells were used for micronuclei and apoptotic analysis. Antiapoptotic effect of ARE (400 mg/kg bw) was measured by the use of Annexin V-FITC assay kit. 4-NQO generated the frequency of micronucleated polychromatic erythrocytes (MnPCEs) by about 4.7 times the control value, 14.29 MnPCEs/2500 PCEs. Pretreatment with ARE significantly reduced the MnPCEs frequency (39–72%) with respect to their doses, and increased PCEs/NCEs ratio was observed over the 4-NQO alone. 4-NQO-induced total apoptotic cells were about 12% over the control which was significantly (*P* < 0.05) brought down to 3.5% by pretreatment with 400 mg/kg bw of ARE. This was the first report that recorded the protective effects of *I. racemosa* on 4-NQO-induced DNA damage and apoptosis in mice bone marrow cells.

## 1. Introduction

Recently, the scientific interest is to develop potential drugs of plant origin for elimination of various diseases including cancer induced by mutagens/carcinogens. It is known that mutations in somatic cells play a key role in cancer initiation and carcinogenesis process [1]. A large number of mutagens have been identified and are known to be potentially deleterious to human health. It is essential to identify bioactive compounds from plants to eliminate these diseases due to human exposure of different environmental mutagens/carcinogens [2, 3]. Many plant extracts have been used as herbal drugs for various toxin-induced diseases in mammals. These drugs have an advantage over the synthetic drugs in terms of low or no toxicity at the effective dose [4]. Medicinal plants are also considered to be dietary substances due to their essential bioactive compounds like vitamin, carotenoids, phenolics, flavonoids, glycosides, organic acids, sterols, some essential oils, and so forth [5]. Therefore, the present study was performed as part of an attempt to authenticate the use of *Inula racemosa* root extract as a traditional medicine in India by investigating their biological properties in an *in vivo* model.

*Inula racemosa* Hook. f. (Asteraceae) is commonly known as Pushkarmoola and grows in the hilly regions of northwestern Himalayas. In India, the plant root powder is used for treating various diseases including cardiovascular disease and as Ayurvedic medicine for angina and dyspnea [6, 7]. Generally, it is a well-known traditional medicine in East Asia and Europe. In China, the root extracts of *I. racemosa* have been prescribed as medicine usually for revitalizing the spleen, regulating the function of the stomach, relieving the depression of the liver qi, alleviating the pain especially between the neck and the shoulders, and to preventing abortion [8]. Root extract was also prescribed as antimicrobial agent and as a tonic in veterinary medicine in different parts of the world [7]. The root extract was studied with equal proportion of *Commiphora mukul* against 200 patients with ischemic heart disease. Moreover, medicinal properties of this plant are mainly due to the presence of bioactive compounds such as alantolactone, isoalantolactone, dihydroalantolactone, dihydroisoalantolactone, sitosterol, daucosterol, inunolide, aplotaxene, phenylacetonitrile, and isoinunal [9, 10]. Medicinal plants are economically important, and besides, it is important to state that the plant has no recorded antimutagenic practice. Therefore, the preliminary study was performed to evaluate the protective effects of aqueous root extract of *I. racemosa* on 4-nitroquinoline-1-oxide induced-DNA damage and apoptosis in mice bone marrow cells.

## 2. Materials and Methods

### 2.1. Animals

 Either sex of* Swiss albino* mice (10–12 weeks old) was obtained from the King Institute, Chennai, India. Animals were maintained at the Departmental Animal House under standard environmental conditions (22 ± 2°C and 12 h light/dark period). Maintenance of animals was done in accordance with the guidelines of the Committee for the Purpose of Control and Supervision of Experiments on Animals (CPCSEA), Government of India. The Institute's ethical committee approved all the experiments.

### 2.2. Plant Material and Chemicals

The roots of *I. racemosa* were collected from the Ramaswamy Country Drug Merchants & Co. Ltd., Chennai, India. The plant root was authenticated by Dr. Sasikala Ethirajulu, Research Officer, Central Research Institute of Siddha, Chennai, India. 

4-Nitroquinoline-1-oxide (4-NQO), Annexin V-FITC assay kit, Giemsa stain, and May-Grunwald stain were purchased from Sigma-Aldrich, USA. 

### 2.3. Aqueous Root Extraction from *Inula racemosa*

The dried roots of *I. racemosa* were chopped into tiny pieces and made into powdery form with an electric blender. Three hundred grams of the powdered root sample of *I. racemosa* was weighed and soaked into 1 L of distilled water for 72 hours at room temperature. The extract was filtered with muslin cloth and then centrifuged. The aqueous extract was freeze-dried and the obtained yield about 20% was stored at −20°C until further use [7]. 

### 2.4. Experimental Design for Antimutagenic Effects of *Inula racemosa*

Either sex of *Swiss albino* was divided into six groups with six mice each. The concentrated aqueous root extract (ARE) was dissolved in sterile distilled water and administered by oral gavage to the mice for 5 consecutive days. Control group 1 was fed with distilled water orally. Group 2 received 400 mg/kg bw of ARE. Group 3 received 7.5 mg/kg bw of 4-NQO intraperitoneally. Groups 4, 5, and 6 received respective test doses 100, 200, and 400 mg/kg bw of ARE for five consecutive days plus 4-NQO (7.5 mg/kg bw; i.p.) on sixth day. After 24 hours of 4-NQO treatment, all the animals were sacrificed by cervical dislocation. The mouse bone marrow assay was carried out by the method of Schmid [11] and their procedure was briefly explained in an earlier publication [12]. For each animal (experimental/control), 2500 polychromatic erythrocytes (with or without micronuclei) and a corresponding number of normal chromatic erythrocytes (NCEs) were scored under a light microscope. 

### 2.5. Experimental Design for Antiapoptosis

The effective antimutagenic dose of ARE was selected to study the antiapoptosis in mice bone marrow cells induced by 4-NQO. The study contained four groups, and in each group, six mice of either sex were used. Group 1 was used as a control which was fed with distilled water orally. Group 2 was fed orally with 400 mg/kg bw of ARE for five consecutive days. Group 3 was injected with 7.5 mg/kg bw of 4-NQO intraperitoneally. Group 4 was fed with ARE plus 4-NQO injected i.p. on the sixth day. After 24 hours, both femurs were removed from the animals under light diethylether anesthesia. The bone marrow cells were flushed out into phosphate buffered saline (PBS) and the cells were prepared for the detection of apoptotic cells based on the protocol reported in our previous publication [12]. 

### 2.6. Statistical Analysis

Results were presented as mean ± standard error for six mice of each group. Statistical analyses were performed by one-way ANOVA using SPSS Software version 12.0. The Student-Newman-Keuls test (SNK test) was applied to assess the differences among the groups. Values of *P* ≤ 0.05 were considered to be significant. 

## 3. Results 

The results on the frequency of micronucleated polychromatic erythrocytes (MnPCEs) for various doses of ARE of *I. racemosa* are presented in Table 1. 4-NQO enhanced the frequency of MnPCEs by about 4.7 times than the control value, 14.29 MnPCEs/2500 PCEs. Irrespective of the doses, treatment with ARE alone did not show significant effect on the frequency of MnPCEs. Pretreatment with ARE significantly reduced the mutation frequency induced by 4-NQO. The reduction was greatest about 72% at the highest dose, 400 mg/kg bw of ARE. The lowest dose of ARE showed about 39% at 100 mg/kg bw. Other doses of 200 mg/kg bw of ARE also significantly modulated 4-NQO-induced MnPCEs. However, the 4-NQO-induced MnPCEs reduced by ARE were found to be dose dependent and consistently significant (*P* < 0.05).

**Figure 1:**
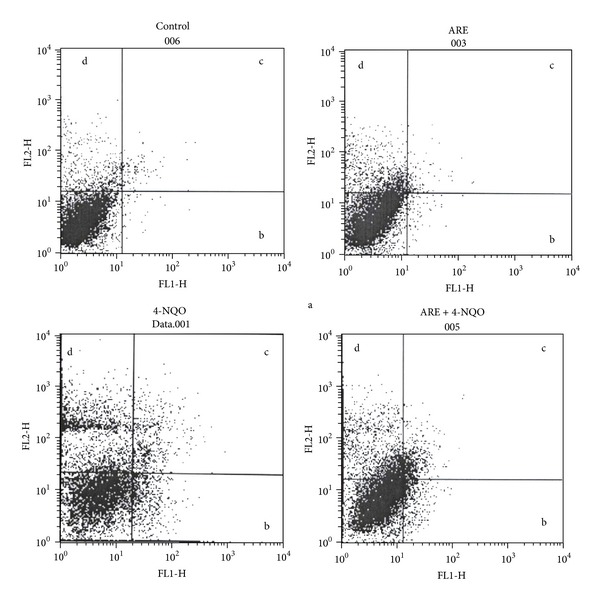
4-NQO-induced apoptosis in mouse bone-marrow cells was measured with annexin V-FITC kit by flow cytometry. (a) Nonapoptotic live cells (annexin V-FITC^−ve^ and PI^−ve^). (b) Early apoptotic cells (annexin V-FITC^+ve^ and PI^−ve^). (c) Late apoptotic and necrotic cells  (annexin  V-FITC^+ve^ and PI^+ve^). (d) Damaged cells (annexin V-FITC^−ve^ and PI^+ve^).

**Figure 2:**
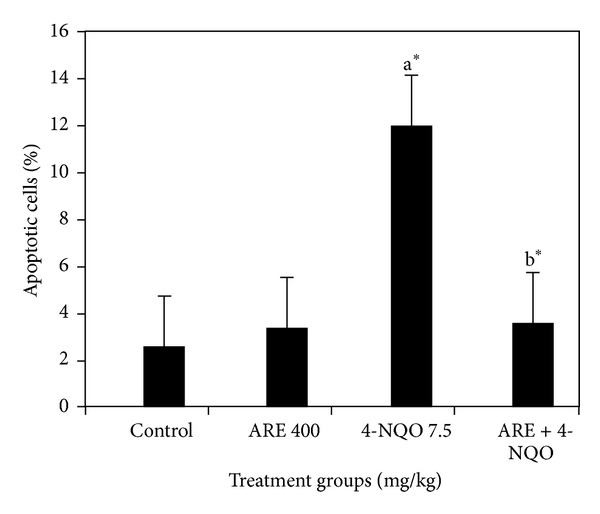
Antiapoptotic effects of aqueous root extract of *I. racemosa* on 4-NQO-induced apoptosis in mouse bone marrow cells. ARE: aqueous root extract. Data were expressed as mean ± standard error (*n* = 6). Significant difference at *P* < 0.001*** and *P* < 0.05* (Student-Newman-Keuls); “a” stands for comparison with the control group; “b” stands for comparison with the only 4-NQO group.

The modulatory effects of ARE on 4-NQO-induced total apoptosis in mice bone marrow cells are exhibited in Figures 1 and 2. Control showed ~2.6% of total apoptotic cells. 4-NQO alone exhibited 12% of total apoptotic cells over the control. Treatment with ARE alone recorded 2.7% of total apoptotic cells, whereas pretreated groups significantly (*P* < 0.05, Figure 2) reduced the total apoptotic bone marrow cells when compared to the control group. There was a significant reduction of apoptotic cells by ARE ~3.5% compared to the 4-NQO alone group. The results revealed that ARE could have the potential to protect the 4-NQO-induced genetic damage.

## 4. Discussion

The present study validated the protective effects of traditional medicine of* I. racemosa* on 4-NQO-induced DNA damage and apoptosis in mice. 4-NQO is a potent mutagen, and during its metabolism, DNA adducts occur through interaction between DNA and 4-NQO intermediate, 4-hydroxyaminoquinoline 1-oxide (Ac-4-HAQO) [13]. As a consequence, the helical structure of DNA changes which results in micronuclei/chromosomal breakage [14]. DNA damage is known to be one of the hallmarks of apoptosis [15]. Due to mutagenic stress, cells undergo apoptosis in the case of DNA damage being beyond repair. Moreover, 4-NQO can induce apoptosis by the p53-dependent mitochondrial signaling pathway [13]. Recently, we reported protective effects of sesame oil on 4-NQO-induced DNA damage in mice [16]. The results revealed that 4-NQO enhanced 4.7-folds of MnPCEs frequency over the control value (*P* < 0.05; Table 1). The reduction of MnPCEs by ARE was found to be 3.6-folds over the 4-NQO alone and also comparable to the control values. The reduction of MnPCEs by ARE was about 39 −72% depending upon the dose. However, the 4-NQO-induced MnPCEs reduced by ARE were consistently significant (*P* < 0.05). PCE/NCE ratio also significantly decreased by about 0.75 in 4-NQO alone than that of control, 1.41. Pretreatment with ARE significantly increased the PCE/NCE ratio in the range between 0.97 and 1.15 over the 4-NQO alone (Table 1). The PCE/NCE results revealed that the ARE possessed a protective potential against 4-NQO-induced genetic damage in mice bone marrow cells. Besides, ARE significantly modulated 4-NQO-induced apoptosis in mice bone marrow cells (Figure 1). The total apoptotic cells enhanced by 4-NQO were found to be 4.6-folds higher than the control value. Pretreatment with ARE-reduced total apoptotic cells was 3.4-folds over the 4-NQO alone group. The result shows that the extract could have the potential to protect the 4-NQO-induced genetic damage in mice bone marrow cells. 

The *in vivo* experiments performed support that the feeding of medicinal plants extracts into mice/rats results in an increase in self-defense mechanisms [4]. *I. racemosa* root also possessed various biological activities such as anti-inflammatory, antimicrobial, and anthelmintic. It is mainly due to the presence of their major bioactive compounds such as sesquiterpene lactones, alantolactone, and isoalantolactone [17]. Antioxidant and cardioprotective effects of *I. racemosa* were recorded [18]. It is also reported to be potential against various types of diabetes [7, 19].

## 5. Conclusion

The present study explored that the root extract of *I. racemosa *could be continued as traditional medicine and also used as natural chemopreventive drug. 4-NQO-induced genetic damage in mice was modulated by ARE of *I. racemosa *via effective restoration of micronuclei and apoptotic cells formations. The potential protective effects might be due to the synergistic effects of secondary metabolites present in ARE of *I. racemosa *[20]. 

## Figures and Tables

**Table 1:** Antimutagenic effect of aqueous root extract of *Inula racemosa* on 4-NQO-induced genetic damage in mice.

Treatment (mg/kg bwt)	MnPCEs/2500 PCEs	PCE/NCE	Decrease (%)
Control (DDW)	14.29 ± 0.32	1.32	—
ARE (400)	13.15 ± 1.05	1.45	—
4-NQO (7.5)	67.12 ± 4.93^a∗∗∗^	0.75	100.00
ARE (100) + 4-NQO	40.71 ± 2.76^b∗∗∗^	0.97	39.35
ARE (200) + 4-NQO	31.93 ± 1.03^b∗∗∗^	1.15	52.43
ARE (400) + 4-NQO	18.56 ± 1.80^b∗∗∗^	1.05	72.35

ARE: aqueous root extract; mean ± standard error (*n* = 6); 4-NQO: 4-nitroquinoline-1-oxide; 2500 PCEs/mice; MnPCEs: micronucleated polychromatic erythrocytes; NCE: normalchromatic erythrocytes; significant difference at ***P < 0.001 and *P < 0.05 (Student-Newman-Keuls); “a” stands for comparison with control group; “b” stands for comparison with 4-NQO alone group.
